# Does the Direct Settlement Policy of Trans-Provincial Outpatient Expenses Aggravate the Siphoning Effect? An Empirical Study on Yangtze River Delta, China

**DOI:** 10.3390/ijerph181910001

**Published:** 2021-09-23

**Authors:** Li Li, Qingyu Zhou, Ting Yin, Zisheng Ji, Lufa Zhang

**Affiliations:** 1 School of International and Public Affairs, Shanghai Jiao Tong University, Shanghai 200032, China; li_li@sjtu.edu.cn (L.L.); ting.yin@sjtu.edu.cn (T.Y.); j295541914@sjtu.edu.cn (Z.J.); 2 Research Institute of Health Development Strategies, Fudan University, Shanghai 200032, China; zhouqingyu@fudan.edu.cn

**Keywords:** direct settlement of trans-provincial outpatient expenses (DSTOE), cross-regional medical treatment, siphoning effects, health insurance

## Abstract

To solve the problem of reimbursing trans-regional medical expenses, using only cross-regional manual reimbursement but not direct medical insurance card settlement, China implemented a pilot policy of direct settlement of trans-provincial outpatient expenses (DSTOE) in the Yangtze River Delta region. Due to the differences in inter-regional medical development, patients often migrate from areas with low-level medical resources to the high-level areas, a phenomenon that we define as the “siphoning” of trans-regional patients, which can cause a variety of problems. To study whether DSTOE aggravates the siphoning effect, we analyzed the changes in the volume of trans-provincial outpatient visits and conducted a questionnaire survey and factor analysis on the willingness of trans-provincial medical treatment under DSTOE. Results showed that manual reimbursement was gradually replaced by direct settlement, while the total volume was not increased significantly, and the ratio of outpatient visits flowing into and out from Shanghai decreased. The majority of questionnaire respondents confessed that their willingness toward trans-regional medical treatment increased, while their first choice of medical location was still mainly local, with only a few indicating that they would directly choose a cross-regional, higher-level medical institution. Spatial accessibility significantly restricted the seeking of trans-regional medical treatment, whereas age, education level, and policy awareness served as significant protective factors for the choice of medical location. In conclusion, due to space accessibility constraints, insufficient policy coverage, and the rationale for choice of location, DSTOE did not aggravate the siphoning effect of trans-regional patients.

## 1. Background

China has put in place the world’s largest social health insurance system, which already covers 1.36 billion people as of 2020 [1], and it has initially completed urban–rural integration [2]. However, since China’s medical insurance pooling level remains at the county and city level, insured residents need to go through a series of troublesome procedures for reimbursement after receiving medical treatment outside their insured area [3]. With the continued urbanization of China [4], the gradual increase in cross-regional population mobility [5] has also increased the demand for medical treatment outside insured areas, which makes reimbursement of medical treatment in different insured areas increasingly troublesome. This situation presents a great challenge for the reform of China’s medical insurance system. In this context, China implemented a pilot policy of direct settlement of trans-provincial outpatient expenses (DSTOE) in September 2018 across the Yangtze River Delta region, which includes the four provincial administrative regions of Shanghai, Jiangsu, Zhejiang, and Anhui [6]. As of 2020, the pilot has expanded to all 41 cities in the Yangtze River Delta and has enrolled 5173 medical institutions [7].

The Yangtze River Delta is the region with the largest scale of trans-provincial medical treatment in China. Among these, Shanghai, a medical highland in the Yangtze River Delta, is a strong attraction for trans-provincial patients, especially those from the Yangtze River Delta. The data show that Shanghai ranks first in China in terms of the influx of trans-provincial patients [8]. Due to the imbalanced distribution of medical resources, a large number of trans-provincial patients visit Shanghai for higher-level medical treatment every year [9], which we call the “siphoning effect” of trans-provincial patients. The siphoning effect has not only exerted unwanted pressure on medical resources in Shanghai by causing problems such as difficulty in registering and long queues in outpatient clinics [10,11], but also caused the loss of patients in their insured areas, which is harmful to the development of local medical services, thereby aggravating the unbalanced distribution of medical resources [12].

The inconvenience of medical treatment reimbursement in different insured areas has always been regarded as a floodgate for trans-provincial medical treatment, which restricts patients from seeking nonessential trans-provincial medical treatment. Since the implementation of the policy of DSTOE, there has been a concern that the facilitation of trans-provincial medical insurance settlement will open the floodgates, further promoting trans-regional patient siphoning in the Yangtze River Delta [13]. However, there is still a lack of evidence to address this concern. As such, we aimed to conduct an empirical analysis on whether the DSTOE policy has intensified trans-regional patient siphoning in the Yangtze River Delta area.

## 2. Methods and Materials

### 2.1. Instruments

To study whether the policy of DSTOE aggravated the siphoning of trans-provincial patients by Shanghai, this study was conducted using three approaches. Firstly, through institutional statistical data, we analyzed the changes that have occurred since the implementation of DSTOE, and whether there has been a significant increase in the total and structural changes in the volume of trans-provincial outpatients around Shanghai and the other three provinces in the Yangtze River Delta. Secondly, through a questionnaire survey, we evaluated the views of people regarding DSTOE after the implementation of the policy on three aspects: their awareness of DSTOE, their willingness to seek trans-provincial medical treatment, and their location choice for the first medical consultation. Thirdly, we also analyzed the factors affecting patients’ trans-provincial medical inclinations, including travel convenience, age, household income, education level, and self-reported health status.

### 2.2. Data Collection

#### 2.2.1. Trans-Provincial Outpatient Volume Data

According to the policies of trans-provincial outpatient settlement, the actual outpatient settlement can be done in three ways: (1) self-pay (outpatient expenses are not reimbursed by insurance), (2) manual reimbursement (patients pay in advance and return to the medical insurance institution of the insured area for reimbursement), and (3) direct settlement with medical insurance card (patients need not return to the insured area for reimbursement).

In this study, we mainly analyzed the volume of outpatient direct settlement in two moving directions, including patients from Shanghai to Jiangsu, Zhejiang, and Anhui (direction A), and patients from Jiangsu, Zhejiang, and Anhui to Shanghai (direction B). To analyze the trans-provincial outpatient volume of both directions, we obtained relevant data from the Shanghai Medical Insurance Center, Jiaxing Medical Insurance Center, and four key tertiary hospitals in Shanghai. The specific data sources are listed in Table 1.

#### 2.2.2. Questionnaire Survey

**Figure 1:**
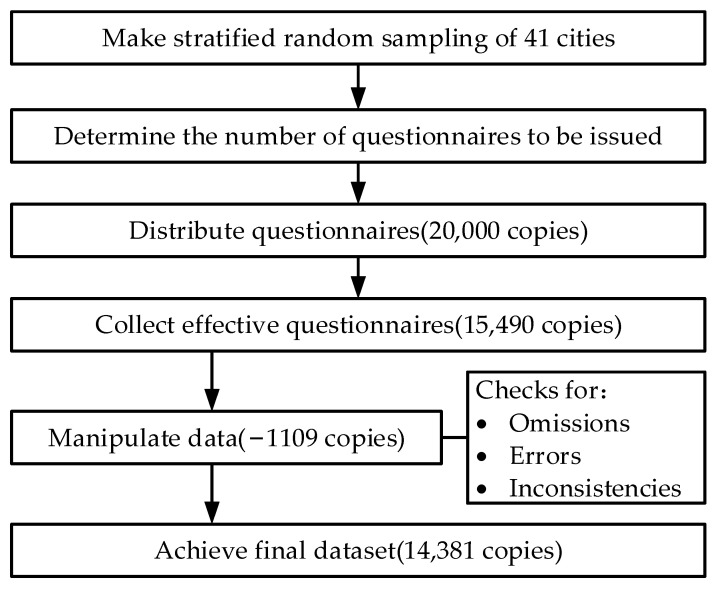
Data collection flowchart.

As shown in Figure 1, according to the established proportions of city, age, and sex, the research team distributed 20,000 copies of the questionnaire regarding trans-provincial medical treatment and the policy of DSTOE, through a stratified random sampling of patients across 41 cities in the Yangtze River Delta. After a total of 15,490 copies were retrieved and 1109 unqualified copies were discarded, a final total of 14,381 valid copies were obtained. The inclusion criteria were (1) respondents who were more than 15 years old, (2) who lived in Shanghai, Jiangsu, Zhejiang, or Anhui province, and (3) who enjoyed one or more types of medical insurance. The exclusion criteria were missing answers or not filling in answers as required.

A descriptive analysis of the social anthropological information in the valid copies is shown in Table 2. Household income (monthly), education level, and health status were self-reported by the respondents. Household income (monthly) refers to the total monthly household income after tax. Education level refers to the final educational background when respondents answered the questionnaire, classified in three levels as high school and below, junior college degree, and bachelor’s degree and above. To establish self-reported health status, respondents were asked the following question: “Would you say your health in general is healthy, fair, or unhealthy?”

### 2.3. Data Analysis

We adopted Pearson’s correlation test to examine the relationship between trans-provincial outpatient volume and the distance to Shanghai, and we drew a heatmap of trans-provincial outpatient volume to Shanghai using Excel map tools (Microsoft Corporation, Redmond, WA 98052-7329, USA). Moreover, we adopted logistic regression analysis to analyze the influence of factors on choice of first consultation place under the policy of DSTOE. All *p*-values were double-sided with a CI of 95%.

## 3. Results

### 3.1. Outpatient Volume of Direct Settlement

**Figure 2:**
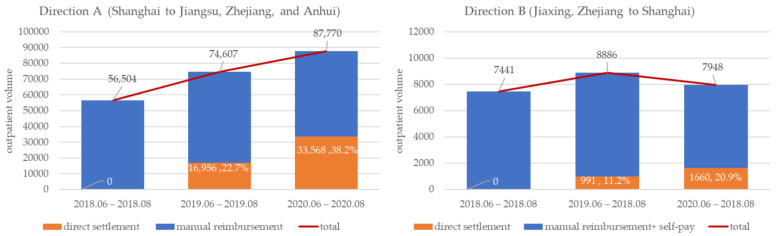
Outpatient volume of direct settlement in two directions.

To ensure the comparability of the analysis and exclude the impact of the COVID-19 epidemic from February to May 2020, we chose three time periods from June to August in 2018, 2019, and 2020, respectively, for comparison. The results showed that, after the implementation of DSTOE, the total number of trans-provincial outpatients in direction A (from 56,504 to 87,770, increased by 55.3%) and the proportion of direct settlement (from 0.0% to 38.2%) increased significantly (Figure 2).

In direction B, taking Jiaxing, Zhejiang as an example (Figure 2), the total number of trans-provincial outpatients initially rose in 2019 (from 7441 to 8886) and then fell in 2020 (from 8886 to 7948) for a net gain of 6.8% in 2 years, while a faster growth in the proportion of direct settlement outpatients was observed (from 0.0% to 20.9%). The results suggest that, after the implementation of the policy, the stimulus effect on the total provincial outpatient volume was not significant, especially in direction B. The trans-provincial outpatient volume indicated more structural changes, such as the gradual replacement of the original manual reimbursement via the direct swiping of medical insurance cards.

**Figure 3:**
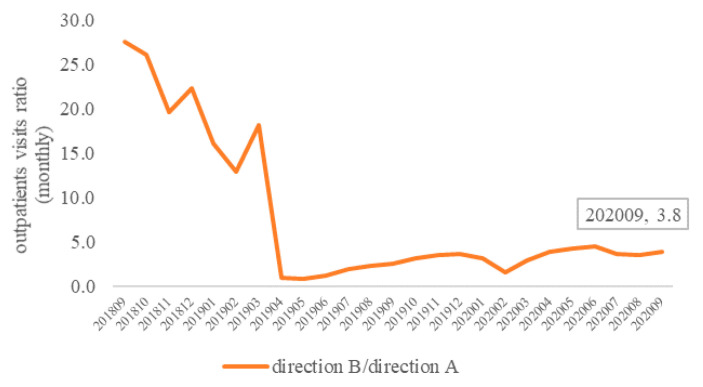
Outpatient visit ratio in both directions (direction B/direction A).

Further analysis was conducted on the ratio of outpatient visits in both directions (direction B/direction A). As shown in Figure 3, in the 2 years of the pilot program (September 2018–September 2020), the ratio of direct settlement for outpatient visits in the two directions gradually decreased and stabilized in the later period. As of September 2020, the ratio of outpatient visits in both directions was 3.8. The visits from three provinces of Jiangsu, Zhejiang, and Anhui to Shanghai accounted for 73% of the total in both directions, while visits from Shanghai to the three provinces accounted for 27% of the total. The above results show that, although the implementation of the policy has attracted more trans-provincial patients to Shanghai for outpatient treatment, there are still a considerable number of Shanghai insured patients going to Jiangsu, Zhejiang, and Anhui for outpatient treatment. The ratio of the two directions was relatively balanced, which suggests that the implementation of the policy did not siphon patients from the insured area to Shanghai as per our predictions.

### 3.2. Questionnaire Survey in 41 Cities of Yangtze River Delta

As shown in Table 3, among the surveyed population, only 6399 (44.50%) were aware of the DSTOE. After being made aware of the policy, 9184 (63.86%) respondents reported an increase in their willingness to seek trans-regional medical treatment, suggesting that the convenience of settling trans-provincial medical expenses has a promoting effect on the willingness to seek trans-provincial medical treatment.

However, when asked about their choice of an initial visit, 7349 (51.10%) of the respondents would still choose to go to local medical institutions first. In addition, 5693 (39.59%) would choose the place of medical treatment according to their actual condition, which meant that they would choose local medical institutions for mild illnesses, and they would only choose to visit medical institutions out of the insured area for better medical treatment if they had a severe illness. Only 1339 (9.31%) respondents would consider going directly to a remote place with better medical conditions for the first medical consultation. The above results suggest that people are still relatively rational in choosing the location for the first consultation, whereby local medical resources are still their first choice, and trans-provincial medical treatment is seen as a last resort to treat serious illnesses that cannot be treated by local medical institutions.

### 3.3. Factor Analysis of Trans-Provincial Medical Treatment Seeking

#### 3.3.1. Spatial Accessibility

Using four key general hospitals in Shanghai as samples, we analyzed the volume of outpatient visits who directly used insurance cards for settlement from 40 cities in the Yangtze River Delta to reflect the attractiveness of Shanghai to insured persons in different cities after the implementation of the DSTOE. To ensure comparability, a timespan was selected from October 2019 to September 2020, while outpatient visits using direct insurance card settlement standardized by city population were used for comparison, which is abbreviated as “standardized outpatient volume (SOV)” (visits/105).

**Figure 4:**
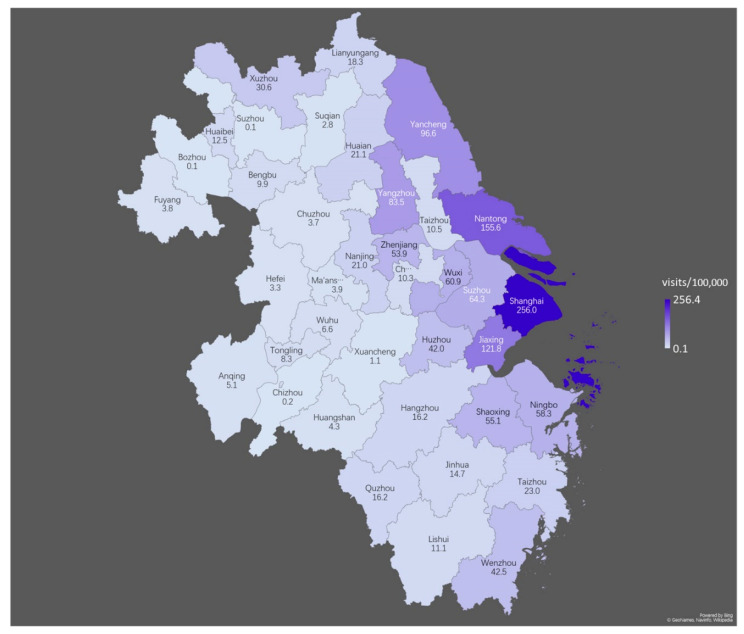
The SOV (visits/100,000) from the 40 cities of Yangtze River Delta to four hospitals in Shanghai (October 2019–September 2020).

The results of the heatmap (Figure 4) show that SOV was much higher in cities around Shanghai, and that the SOV decreased as the distance from Shanghai increased. Pearson correlation analysis results showed a significant correlation between SOV and the distance to Shanghai, with a correlation coefficient of −0.491 (*p* = 0.001, two-tailed) (Supplementary Table S1). The results above suggest that the attraction of Shanghai to trans-provincial patients is significantly restricted by the accessibility of space.

**Figure 5:**
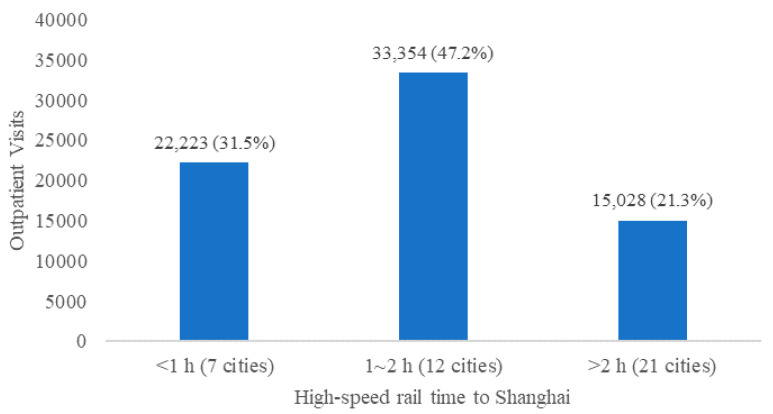
The volume of trans-provincial outpatient visits from the 40 cities of Yangtze River Delta to four hospitals in Shanghai. Note: data are classified according to the shortest time to Shanghai by high-speed rail; October 2019–September 2020.

We classified the 40 cities according to the number of high-speed rail hours taken to reach Shanghai (Figure 5) and found that the outpatient visits with direct insurance card settlement (OV) within 1 h (7 cities) of Shanghai accounted for 31.5%, OV within 1 h to 2 h (12 cities) to Shanghai accounted for 47.2%, and OV of the other 21 cities more than 2 h away from Shanghai accounted for only 21.3%, which further supports the impact of spatial accessibility on trans-provincial outpatients.

#### 3.3.2. Determinants of Choice of Location for the First-Medical Consultation

It is considered a reasonable and rigid demand that patients are transferred to medical institutions with better conditions out of town when local medical resources are insufficient to meet the needs of disease treatment. This is the kind of demand that the DSTOE policy aims to serve. In contrast, the direct choice of first diagnosis in medical institutions outside the insured area, induced by the convenience of trans-provincial reimbursement under DSTOE, is a nonrigid pursuit of high-level medical treatment, which deviates from the principle of hierarchical diagnosis and treatment [14]. This kind of excessive trans-regional medical treatment would undoubtedly bring unnecessary pressure to medical institutions in the medical treatment area, which policymakers hope to control.

According to whether the respondent would directly choose medical institutions out of the insured area under the DSTOE policy, we divided the choice of first medical consultation into two categories. We then adopted logistic regression to analyze the effects of factors on the choice of first consultation place under the policy, such as age, family income, education level, self-reported health level, and policy awareness.

As shown in Table 4, compared with younger respondents (age 15–49), aging respondents (age > 65) were less likely to choose a cross-regional institution for the first consultation (β = −0.402, OR = 0.669, *p* < 0.001). Respondents who self-reported as healthy were more likely to seek trans-regional medical consultation, and this tendency was 1.464 times that of respondents who self-reported as unhealthy (β = 0.381, OR = 1.464, *p* = 0.006). Interestingly, respondents who were aware of the policy were 30.4% less likely to choose trans-regional consultation than those who were not (β = −0.363, OR = 0.696, *p* < 0.001).

## 4. Discussion

### 4.1. Non-Aggravation of Trans-Provincial Patient Siphoning in the Yangtze River Delta by DSTOE

From the perspective of changes in the volume and structure of outpatients after the implementation of the policy, the total number of outpatient clinics in the Yangtze River Delta did not increase significantly, while the direct settlement rate continued to rise, mainly due to structural adjustments. Moreover, judging by the proportion of trans-provincial outpatients with direct settlement in the two directions, the DSTOE pilot has met the medical needs of not only the trans-provincial outpatient patients from Jiangsu, Zhejiang, and Anhui to Shanghai, but also vice versa to a large extent. It can be said that the convenience of trans-provincial medical insurance settlement has not caused increased unidirectional outpatient flows in the Yangtze River Delta. In other words, there is no evidence of a cross-regional siphon. In general, the DSTOE policy has achieved mutual benefit between Shanghai and the three provinces of Jiangsu, Zhejiang, and Anhui, and an increasing number of patients with actual trans-provincial medical needs have enjoyed the convenience brought about by the DSTOE policy.

### 4.2. Analysis of the Reasons Why Siphoning of Trans-Provincial Patients Was Not Caused by DSTOE

First, trans-provincial outpatient visits were significantly restricted by the convenience of travel. Since outpatient expenses are generally small, travel costs, which cannot be reimbursed by medical insurance, can account for a considerable proportion of the total expenses of trans-provincial outpatient treatment. This makes travel convenience an important factor in the choice of outpatient locations [9,15]. Our results showed that outpatient visits with direct insurance card settlement from 40 cities in Jiangsu, Zhejiang, and Anhui to Shanghai showed a significant negative correlation with the distance from the insured cities to Shanghai, which also supports the restrictive effect of travel costs on trans-provincial outpatient treatment. Therefore, even with the convenience increase in medical insurance settlement for trans-provincial outpatients, the attractiveness of high-level medical areas such as Shanghai to trans-provincial patients is still limited. A longer distance leads to lower attractiveness, which makes the scope of the siphon effect controllable.

Second, the patients’ location choice for first medical consultation was still rational. From the analysis of the questionnaire results, most of the interviewees would still choose their local institution for the first medical consultation even when aware of the DSTOE policy, and they would only consider going to a cross-regional institution with better medical resources if their symptoms were severe, which indicates a clear relationship between the location choice of the first medical consultation and the severity of the patient’s health condition. Generally, patients with diseases that can be effectively resolved locally would not choose to seek trans-provincial medical treatment. Therefore, we can consider that the population seeking trans-provincial medical treatment is controllable.

Lastly, the low policy awareness rate was also one of the reasons why DSTOE did not aggravate the siphoning of trans-provincial patients. After all, our results showed that the policy awareness rate was only 44.5%. This might be due to the conservative propaganda principles adopted by government departments. Through further interviews after the questionnaire survey, it was found that most interviewees were aware of the DSTOE policy only through hospitals and medical insurance reimbursement agencies. In addition, as shown in our survey, there are still many diseases, such as outpatient chronic diseases, outpatient special diseases, and other diseases with higher demands and higher costs, not included in the scope of DSTOE due to large policy differences across cities [16]. These kinds of problems have also been corroborated in other studies [17,18].

### 4.3. Policy Recommendations

At present, this policy has entered a critical period, from pilot to nationwide promotion. After the Fourth Session of the 13th National People’s Congress, Premier Li Keqiang claimed that, by the end of 2022, each county will enroll at least one designated medical institution that will directly reimburse medical expenses, including outpatient fees [19]. To prevent the promotion of DSTOE stimulating the siphoning of trans-provincial patients, we recommend that efforts be directed in two aspects.

First, we recommend implementing a policy of cross-regional hierarchical diagnosis and treatment to promote the orderly flow of cross-regional patients. The results of the questionnaire survey showed that 63.86% of the respondents still had an increased willingness to seek better trans-regional medical treatments under the policy, which indicates that we are still facing the risk of the policy triggering the siphoning of trans-regional patients. Just as floods should be dredged rather than blocked, we recommend implementing a cross-regional hierarchical diagnosis and treatment in the four provinces of the Yangtze River Delta, which means that they should visit their local primary hospital for first medical consultation, seek treatment at local higher-level medical institutions for common diseases and most intractable diseases, and visit regional and even national medical centers through more convenient channels for a small number of very complicated, difficult, and severe diseases [7]. Through this kind of cross-regional hierarchical diagnosis and treatment, most patients would be kept in local medical institutions, enabling a measure of control over the siphoning of trans-regional patients.

Second, we recommend appropriately increasing awareness of the DSTOE policy, especially for key populations in trans-provincial medical treatment. This study found that policy awareness is not an expected promotion factor for trans-provincial medical treatment, but a protective factor. The public’s understanding of the policy will not stimulate patients to choose trans-regional institutions for the first medical consultation. In contrast, inadequate policy publicity will prevent some patients with reasonable cross-provincial medical needs from successfully swiping their insurance cards, especially for the elderly who might not readily accept the new policy due to their digital illiteracy [20]. As Premier Li Keqiang emphasized, the elderly population is growing, and it has become the norm for them to live with their children outside their hometowns to help out with childcare. Moreover, the elderly have more outpatient needs because they are at higher risk of common chronic diseases and senility; therefore, they are inevitably the largest group seeking outpatient medical treatment in different places [21,22]. On the basis of the above, we recommend targeting publicity efforts at the elderly, which can help us achieve twice the result with half the effort.

## 5. Limitations

It must be confessed that, from January 2020 to June 2020, the COVID-19 epidemic has severely affected trans-provincial medical treatment seeking. In this study, we took some measures to control its impact, such as choosing the time period (July–September) less affected by the epidemic for comparison. However, the bias induced by the epidemic cannot be completely eliminated. In the future, we will continue to track the progress of DSTOE, in order to obtain more evidence.

## 6. Conclusions

Due to space accessibility constraints, insufficient policy coverage, and the rationale in choosing the location for the first medical consultation, DSTOE did not aggravate the siphoning effect of trans-regional patients in Yangtze River Delta. To further prevent the promotion of DSTOE stimulating the siphoning of trans-provincial patients, we recommend implementing a policy of cross-regional hierarchical diagnosis and treatment, as well as appropriately increasing awareness of the DSTOE policy, especially for key populations in trans-provincial medical treatment.

## Figures and Tables

**Table 1:** Trans-provincial outpatient volume data source.

Moving Directions	Outpatient Settlement Method	Data Source
Shanghai to Jiangsu, Zhejiang, and Anhui (Direction A)	Direct settlement	Shanghai Medical Insurance Center
	Manual reimbursement	Shanghai Medical Insurance Center
Jiangsu, Zhejiang, and Anhui to Shanghai (Direction B)	Direct settlement (total)	Shanghai Medical Insurance Center
	Direct settlement + manual reimbursement + self-pay (Sample 1)	four key general hospitals in Shanghai
	Direct settlement + manual reimbursement (Sample 2)	Medical Insurance Center of Jiaxing, Zhejiang

**Table 2:** Statistics of demographic characteristics.

Characteristic	Subcategory	Respondents (%)
Age (in years)	15–29	3211 (23.16%)
	30–49	5439 (42.86%)
	50–64	3612 (22.50%)
	65+	2119 (11.48%)
Sex	Male	7284 (50.65%)
	Female	7097 (49.35%)
Province	Shanghai	3733 (25.96%)
	Jiangsu	3598 (25.02%)
	Zhejiang	3327 (23.13%)
	Anhui	3723 (25.89%)
Household income (monthly)	0–10,000	2900 (20.17%)
	10,000–20,000	7405 (51.49%)
	20,000+	4076 (28.34%)
Education level	High school and below	6063 (42.16%)
	Junior college degree	4764 (33.13%)
	Bachelor’s degree and above	3554 (24.71%)
Self-reported health status	Healthy	10,140 (70.51%)
	Fair	3244 (22.56%)
	Unhealthy	997 (6.93%)

**Table 3:** Questionnaire survey around trans-provincial medical treatment.

Questions	Answers	Volume (%)
Do you know that you can directly use your medical insurance card as an inter-province outpatient?	Yes	6399 (44.50%)
	No	7982 (55.50%)
What has happened to your willingness to seek medical treatment in another city after the policy?	Increased	9184 (63.86%)
	Constant	4641 (32.27%)
	Reduced	556 (3.87%)
Now that you know the policy, how will you choose the location for the first medical consultation?	Prioritizing the place of residence	7349 (51.10%)
	According to the severity of the disease (in the place of residence for milder and in another city with better medical conditions for severer disease)	5693 (39.59%)
	Prioritizing another city with better medical conditions	1339 (9.31%)

**Table 4:** Factor analysis on choice of first consultation place under the policy.

Characteristic	Subcategory	Choose a Better Place with Better Medical Condition When First Outpatient Visit		Logistic Regression		
		No	Yes	β	OR	*p*-Value
Age (in years)	15–49 ^a^	7779 (89.93%)	871 (10.07%)			
	50–64	3298 (91.31%)	314 (8.69%)	−0.193	0.824	0.012
	65+	1964 (92.69%)	155 (7.31%)	−0.402	0.669	<0.001
Household income (monthly)	0–10,000 ^a^	2673 (92.17%)	227 (7.83%)			
	10,000–20,000	6602 (89.16%)	803 (10.84%)	0.262	1.300	0.001
	20,000+	3766 (92.39%)	310 (7.61%)	−0.108	0.898	0.265
Education level	High school and below ^a^	5507 (90.83%)	556 (9.17%)			
	Junior college degree	4307 (90.41%)	457 (9.59%)	−0.177	0.837	0.019
	Bachelor’s degree and above	3227 (90.80%)	327 (9.20%)	−0.167	0.846	0.058
Self-reported health status	Unhealthy	932 (93.48%)	65 (6.52%)			
	Fair	3030 (93.40%)	214 (6.60%)	−0.071	0.931	0.635
	Healthy	9079 (89.54%)	1061 (10.46%)	0.381	1.464	0.006
Policy awareness	Not aware ^a^	5918 (92.48%)	481 (7.52%)			
	Aware	7123 (89.24%)	859 (10.76%)	−0.363	0.696	<0.001

^a^ Group refers to the reference group in the study characteristics in the logistic regression.

## Data Availability

The datasets used and/or analyzed during the present study are available from the corresponding author on reasonable request.

## Supplementary Materials

The following are available online at https://www.mdpi.com/article/10.3390/ijerph181910001/s1: Table S1, Correlation analysis between distance from Shanghai (km) and the outpatient visits (October 2019–September 2020).

## References

[1] (2021). The Communist Party of China and Human Rights Protection—A 100-Year Quest. China: The State Council Information Office of the People’s Republic of China. http://english.scio.gov.cn/whitepapers/2021-06/24/content_77584416.htm.

[2] Zhu K., Zhang L., Yuan S., Zhang X., Zhang Z. (2017). Health financing and integration of urban and rural residents’ basic medical insurance systems in China. Int. J. Equity Health.

[3] Shan L., Zhao M., Ning N., Hao Y., Li Y., Liang L., Kang Z., Sun H., Ding D., Liu B. (2018). Dissatisfaction with current integration reforms of health insurance schemes in China: Are they a success and what matters?. Health Policy Plan..

[4] Gu C.L., Hu L.Q., Ian G.C. (2017). China’s Urbanization in 1949–2015: Processes and Driving Forces. Chin. Geogr. Sci..

[5] Taylor J.R. (2015). The China dream is an urban dream: Assessing the CPC’s national new-type urbanization plan. J. Chin. Political Sci..

[6] (2018). Yangtze River Delta Implements a Pilot Policy of Direct Settlement of Trans-Provincial Outpatient Expenses.

[7] Zhang L.F. (2021). Strategy and pathway for the policy of direct settlement of trans-provincial outpatient expenses—take the Yangtze River Delta region as an example. Nanjing Soc. Sci..

[8] (2021). Announcement of the General Office of the National Health Commission on the National Monitoring and Analysis of the Performance Appraisal of the National Tertiary Public Hospitals in 2019.

[9] Fu L., Xu K., Liu F., Liang L., Wang Z. (2021). Regional Disparity and Patients Mobility: Benefits and Spillover Effects of the Spatial Network Structure of the Health Services in China. Int. J. Environ. Res. Public Health.

[10] Teng X.M., Liao Z.D., Cheng P.R., Chen S. (2020). Study on the Cross Provincial Outpatient Network Settlement in Yangtze River Delta 1Year after the Implementation. Chin. Prim. Health Care.

[11] Wang L.N., Li F., Wang C.Y., Chen W., Xie Z.H., Jin C.L. (2014). Analysis on the impact of non-residents’ medical service utilization on medical services system of Shanghai. Chin. Health Resour..

[12] Yan X., Dong D., He S. (2020). Examining trans-provincial diagnosis of rare diseases in china: The importance of healthcare resource distribution and patient mobility. Sustainability.

[13] Zhang Y., Zheng X.P., Liu Y., Zhang Y.J. (2015). The Problems and Countermeasures of Floating Population’s Medical Insurance Settlement. Health Econ. Res..

[14] Fu Q. (2015). Strategic Choice for Promoting Hierarchical Treatment Model. Chin. Health Econ..

[15] Ulrike S., Jeanette B., Daniel F., Jens P., Wolfgang H., Neeltje B. (2018). Is there an association between spatial accessibility of outpatient care and utilization? Analysis of gynecological and general care. BMC Health Serv. Res..

[16] Lv D.W., Li L., Yin T., Ji Z.S., Xu H., Wang W.J., Li M.Z., Zhang L.F. (2021). A preliminary study on the implementation effect of the inter-provincial direct settlement policy for outpatient expenses in the Yangtze River Delta—Taking Shanghai data as an example. Chin. Med. Insur..

[17] Li C.Y., Gao X.G. (2018). Problems and countermeasures in the promotion of trans-provincial medical treatment. Zhongguo Renliziyuan Shehui Baozhang.

[18] Jiang L.W., Liu C.H., Jiang H., Li B. (2020). Study on Development Status and Problems of Real-time Settlement of Cross-provincial Medical Insurance in China. Chin. J. Soc. Med..

[19] (2021). Li: Reimbursement of Medical Bills to Be Made Easier. China: Chinadaily.com.cn. https://www.chinadaily.com.cn/a/202103/11/WS6049f9dba31024ad0baae970.html..

[20] Neves B.B., Waycott J., Malta S. (2018). Old and afraid of new communication technologies? Reconceptualising and contesting the ‘age-based digital divide. J. Soc..

[21] Fu Y., Lin W., Yang Y., Du R., Gao D. (2021). Analysis of diverse factors influencing the health status as well as medical and health service utilization in the floating elderly of China. BMC Health Serv. Res..

[22] Han K., Yao J., Yin X., Zhao M., Sun Q. (2017). Review on the prevalence of diabetes and risk factors and situation of disease management in floating population in China. Glob. Health Res. Policy.

