# Supplementary material for: Does the Direct Settlement Policy of Trans-Provincial Outpatient Expenses Aggravate the Siphoning Effect? An Empirical Study on Yangtze River Delta, China
Source: Int J Environ Res Public Health. 2021 Sep 23;18(19):10001. doi: 10.3390/ijerph181910001 (PMC8507957; doi:10.3390/ijerph181910001)
Supplement: Supplementary file 1 [file ijerph-18-10001-s001.zip › ijerph-1345597-supplementary.pdf]

International Journal of
Environmental Research and Public Health

MDPI

Supplementary

Table S1. Correlation analysis between distance from Shanghai (km) and the outpatient visits (October 2019–September 2020).

Province	Cities	Visits/10^5*^	Distance from Shanghai(km)**	Pearson's Correlation	
				Coefficient	P-value
**Anhui**	**Anqing**	**5.1**	430.6	-0.491	0.001
Anhui	Bengbu	9.9	233.1		
Anhui	Bozhou	0.1	431.6		
Anhui	Chizhou	0.2	696.0		
Anhui	Chuzhou	3.7	326.1		
Anhui	Fuyang	3.8	174.6		
Anhui	Hefei	3.3	350.4		
Anhui	Huaibei	12.5	383.6		
Anhui	Huainan	0.9	282.6		
Anhui	Huangshan	4.3	494.0		
Anhui	Liuan	6.1	479.4		
Anhui	Maanshan	3.9	464.2		
Anhui	Suzhou	0.1	569.0		
Anhui	Tongling	8.3	361.5		
Anhui	Wuhu	6.6	548.1		
Anhui	Xuancheng	1.1	510.8		
Jiangsu	Changzhou	10.3	608.8		
Jiangsu	Huaian	21.1	479.6		
Jiangsu	lianyungang	18.3	177.1		
Jiangsu	nanjing	21.0	149.2		
Jiangsu	nantong	155.6	127.5		
Jiangsu	Suzhou	64.3	98.5		
Jiangsu	Suqian	2.8	623.8		
Jiangsu	taizhou	10.5	339.5		
Jiangsu	wuxi	60.9	103.7		
Jiangsu	xuzhou	30.6	455.7		
Jiangsu	yancheng	96.6	99.8		
Jiangsu	yangzhou	83.5	226.3		
Jiangsu	zhenjiang	53.9	584.2		
Zhejiang	hangzhou	16.2	372.8		
Zhejiang	huzhou	42.0	297.3		
Zhejiang	jiaxing	121.8	307.9		
Zhejiang	Jinhua	14.7	407.0		
Zhejiang	Lishui	11.1	482.7		
Zhejiang	Ningbo	58.3	275.7		
Zhejiang	Quzhou	16.2	411.4		
Zhejiang	Shaoxing	55.1	248.9		
Zhejiang	Taizhou	23.0	392.1		
Zhejiang	Wenzhou	42.5	131.7		
Zhejiang	Zhoushan	256.4	196.0		

* Visits refers to the outpatient visits to the four hospitals in Shanghai which was directly paid through medical insurance cards from other cities. ** Distance from Shanghai (km) refers to the distance from the city to Shanghai by vehicle.
